# Matrix metalloproteinase gene polymorphisms and oral cancer

**DOI:** 10.4317/jced.50859

**Published:** 2012-12-01

**Authors:** Andresa C. Pereira, Elaine Dias do Carmo, Marco A. Dias da Silva, Luiz E. Blumer Rosa

**Affiliations:** 1 Academic Unit of Biological Sciences, School of Dentistry, Federal University of Campina Grande (UFCG), Patos-PB, Brazil; 2 Health Institute, Faculdades Metropolitanas Unidas (FMU), São Paulo-SP, Brazil; 3 Department of Biosciences and Oral Diagnosis, São José dos Campos Dental School, São Paulo State University (UNESP), São José dos Campos-SP, Brazil

## Abstract

Since oral squamous cell carcinoma (OSCC) is the most prevalent malignant cancer in the oral cavity, several researches have been performed to study the role of important enzymes in this disease. Among them, the matrix metalloproteinases (MMPs) are highlighted, due to the fact that they are proteinases responsible to degrade many extra-cellular matrix components, making possible the invasion of neoplasic cells. Important tools in cancer prognosis have been utilized aiming to correlate high levels of MMPs and OSCC, such as immunohistochemical, zymographic and mRNA detection methods. However, these techniques are usually applied after cancer detection, characterizing a curative but not a preventive medicine. Trying to make interventions before the development of the disease and making possible the identification of people at high risk and, analysis of modifications in MMP genes has been a chance for modern medicine. Recently, polymorphisms in MMP genes have been related to different neoplasias, including OSCC. Despite investigation is beginning, MMP gene polymorphisms seems to have a promising future in oral cancer research and some of the present results have shown that there are MMP polymorphisms related to an increased risk for developing oral cancer.

** Key words:**Oral cancer, polymorphism, matrix metalloproteinase.

## Introduction

For years, research groups have been working on evaluating the presence, activity, function and regulation of matrix metalloproteinases (MMPs) in healthy and diseased oral tissues (1). Today, a new era in oral health professional education is the study and application of human genetic variance during normal development, diseases, disorders, and responses to treatments and therapeutics (2).

- The Matrix Metalloproteinases (MMPs)

The MMPs group is represented by more than 20 human enzymes (3,4) which require presence of zinc to realize their functions. These proteases are classified based not only on the structural domain and organization, but also on the substance that can be degraded by them (5). The main MMP classes are: collagenases, gelatinases, stromelysins, and the membrane-type MMPs (MT-MMP) (6).

The collagenases are the MMPs-1, -8 and -13, known as collagenases 1, 2 and 3 respectively, mainly degrading type I, II and III collagen (4,7). In addition, the MMPs-2 and -9, also called gelatinases A and B respectively, degrade preferentially gelatin (denatured collagen) and type IV collagen (4,8) while the stromelysins 1 and 2 (MMPs-3 and -10) are able to act on non collagen matrix proteins, such as fibronectin (7,8). Finally, the last subgroup, the MT-MMPs, is numbered from 1 to 6: MT1-MMP, MT2-MMP, MT3-MMP, MT4-MMP, MT5-MMP and MT6-MMP, corresponding in order to MMPs-14, -15, -16, -17, -24 and -25. The MT-MMPs present an important action on the activation of other MMPs and each subtype is responsible for the degradation of different extra-cellular matrix components (4,8).

Usually health tissues present low expression of activated MMPs (6), in contrast, an increase in MMPs expres-sion has been related to malignant neoplasias, demonstrating noticeable significance in proteolytic action during the invasion and metastasis processes (4-6).

## Oral Cancer and MMPs tissue levels

Oral squamous cell carcinoma (OSCC) is the most prevalent malignant cancer in oral cavity (9). Despite the histopathology is being the most frequent technique used by pathologists to identify, analyze and rank the clinical phenotype of human cancers, this method may present reservations. Multiple patients with squamous cell carcinoma may provide biopsy samples that closely resemble one another; however, they may present different neoplasic disease, responses to therapy and clinical outcomes (2).

Trying to complete the diagnosis, the MMP detection in the tissue has been investigated using techniques such as immunohistochemistry (10,11), zymography (10,12) and RNA analysis (13).

Studies have shown that OSCC cases seem to express higher levels of different MMPs when compared to normal tissues (13).

The MMPs expression in oral cancer tissue is an important tool in cancer prognosis. However, MMP expression only became substantial if the tests are performed after cancer initiation. Due to this fact, this methodology might be used for therapeutic purposes, but not as a preventive tool, which is the idea of new medicine.

To proteins be expressed in the tissue, their codification must be done based on information coming from the DNA code. If there is an alteration in the gene, it is possible to have a modification in this process, resulting in different MMP protein expressions in the tissue. Thus, the genetic control is particularly important in these regulations and if alterations, as a gene polymorphism, are found, they might determine a different cancer clinicopathological development.

## Oral cancer and MMPs gene polymorphisms

Genotyping MMPs as potential markers for susceptibility to oral cancer might allow a precise and early identification of individuals at high risk. These findings may help not only prediction and prevention, but also the plan of therapeutic methods and evaluation of treatment outcome (14)

Despite numerous genetic studies have correlated MMP gene polymorphisms and several categories of neo-plasias (15-24), few authors have studied the link between MMP gene polymorphisms and oral cancer development (9,14,25).

The genetic polymorphisms are characterized by a modification in the DNA base pairs sequence in more than 1% of the human population. The majority of these alterations are probably functionally neutral; however, the polymorphism may be linked on the regulation of gene expression or function of the coded protein influencing different biological behaviors and susceptibility to diseases (3). Approximately, ninety percent of DNA polymorphisms are single nucleotide polymorphisms (SNP) due to single base exchange.

In the gene, the region responsible for the transcription control is the gene promoter, located upstream from transcription start site and contains sequences for the binding of critical transcriptional factors. In recent times, several DNA polymorphisms were found in the promoter region of various MMPs (3). Particularly in oral cancer, the studies are related to MMP-1, -2, -3 and -9 polymorphisms.

- MMP-1 gene polymorphism

There is a MMP-1 genetic polymorphism which is a promising candidate for susceptibility to cancers (26). This polymorphism in MMP-1 gene promoter is characterized by insertion/deletion of a guanine at position –1607 and formation of two different alleles, one having a single guanine (1G) and the other having two guanine (2G) (27). The cells expressing the 2G polymorphism seem to present a more aggressive matrix degradation system, possibly facilitating cancer development (27). The frequency of the 2G/2G genotype is significantly higher in patients presenting colorectal cancer (16), renal cell carcinoma (19) and head and neck squamous cell carcinoma (28) than in controls.

In addition, the presence of this polymorphism (2G) has been associated not only with an increased risk of local invasiveness in cutaneous malignant melanoma (15) and cervical cancer (22), but also with worse cancer specific survival in colorectal cancer (29) and the risk for the development of metastasis in breast cancer (24).

In oral cancer, the MMP-1 gene was investigated by Lin et al. (14) and Cao and Li (9), who have correlated the Insertion/Deletion (-1607 2G/1G) polymorphism in the promoter region of this gene and the OSCC. Lin et al. (14) concluded that 2G genotype in MMP-1 promoter could be a risk factor for developing OSCC, since this polymorphism was observed with a higher frequency in OSCC cases compared with controls. Similarly, Cao and Li (9) verified that an increased prevalence of the MMP-1 2G/2G genotype in patients with OSCC indicates that this MMP-1 polymorphism confers an increased risk for OSSC. The results showed that the individuals with 2G/2G genotype seem to be 2.5 times more likely to develop the OSCC than those who were 1G/2G or 1G/1G. Nevertheless, none of these studies (9,14) have found statistically significant association between MMP-1 genotype in OSCC patients and clinicopathological parameters.

- MMP-2 gene polymorphism

In MMP-2 gene, a functional single nucleotide, cytosine (C) to thymine (T), polymorphism (-1306 C/T) has been reported (30). It seems that CC genotype may be associated with the high transcriptional and enzyme activity of MMP-2, and eventually affect individual susceptibility to neoplasms (31). Subjects with the CC genotype have been related to increased risk for developing lung cancer (32), gastric cardia adenocarcinoma (33) and breast cancer (20) compared with those with the CT or TT genotype.

Similarly, Lin et al. (31) have verified that patients carrying CC genotype also present increased risk for devel-oping oral cancer when comparing with CT or TT genotype. However, the genotype CC was not correlated to advanced clinical stage of OSCC cases.

- MMP-3 gene polymorphism

A variant in the promoter of the MMP-3 gene has been identified (34) and characterized by one allele having five adenosines (5A) and the other allele having six adenosines (6A), located at position -1171 (35). The 5A allele seems to present a higher promoter activity than the 6A allele (35) and in breast cancer the frequency of 5A allele is likely to be higher in patients than in controls (17). Furthermore, the presence of 5A genotype may be linked to a higher risk for metastasizing among breast cancer (17,36) and esophageal squamous cell carcinoma patients (37). In addition, smokers with at least one 5A allele have a significantly increased risk of esophageal squamous cell carcinoma, compared with 6A homozygotes (37).

However, in oral cancer, the study of the functional nucleotide polymorphism of MMP-3 promoter (-1171 5A/6A) showed that this polymorphism is not a risk factor for oral carcinogenesis (25). Similarly, it was not found significant difference for the MMP-3 genotype in patients with renal cell carcinoma (19), esophageal squamous cell carcinoma (37) and gastric cardiac adenocarcinoma (37) when compared to controls individuals.

- MMP-9 gene polymorphism

It was reported a functional C to T single nucleotide polymorphism at position -1562 in

the MMP-9 promoter, where the T allele was described to have a higher activity than the C allele (38). This DNA modification has been studied in different cancers, such as breast (18,24), gastric (21) and renal carcinoma (23); however, the results are still controversial. In gastric cancer, T allele in the MMP-9 promoter is associated with the invasive phenotype (21), on the other hand, this polymorphism may not be associated with the occurrence of breast (18,24) and renal cancer (23).

In oral oncogenesis, it was reported that the MMP-9 polymorphism (-1562 C/T) presents a strong association with increased risk for developing oral cancer in a subset of the general population (39). The higher gene expression of MMP-9 was conferred by the T allele and the carriers of this nucleotide presented an increased risk for developing OSCC only in initial stages, but not in advanced ones (39).

Investigating whether this MMP-9 polymorphism could influence the risk of areca associated oral cancers, it was seen that this DNA modification was linked with OSCC risk only in younger areca chewers (40). These results illustrate the great importance of genetic association studies, which may result in preventive measures safeguarding the health status and lives of some individuals at risk in the general population (39).

- MMPs research expectations

It is important to say that the MMP gene polymorphisms research is certainly beginning and some results are still controversial due to some important different factors as race, age and type of cancer. Despite these facts, it is believed that in the future, analyses using polymorphisms, will not only identify individual variations within disease comparisons, but will also identify human genetic variations, such as how each person responds to certain therapy. Therefore, this can intensely improve the understanding of risk factors, chemoprevention, diagnosis and treatments of oral cancer (2).

## Conclusion

To sum up briefly, many researches have been aiming to understand the real role of MMPs, phenotypic and genotypically, in oral cancer progress. One important tool is the DNA analysis, whereby the researchers have investigated the hypothesis that MMPs gene polymorphisms might be related to oral cancer development. Although further studies are needed, it is aspired that, in the future, it could be possible to find an oral cancer high risk individual and try to predict the disease development.
